# Arabidopsis RPT2a, 19S Proteasome Subunit, Regulates Gene Silencing via DNA Methylation

**DOI:** 10.1371/journal.pone.0037086

**Published:** 2012-05-16

**Authors:** Kaori Sako, Yuko Maki, Tomoyuki Kanai, Eriko Kato, Shugo Maekawa, Shigetaka Yasuda, Takeo Sato, Masaaki K. Watahiki, Junji Yamaguchi

**Affiliations:** Faculty of Science and Graduate School of Life Science, Hokkaido University, Sapporo, Japan; University of Leeds, United Kingdom

## Abstract

The ubiquitin/proteasome pathway plays a crucial role in many biological processes. Here we report a novel role for the Arabidopsis 19S proteasome subunit RPT2a in regulating gene activity at the transcriptional level via DNA methylation. Knockout mutation of the *RPT2a* gene did not alter global protein levels; however, the transcriptional activities of reporter transgenes were severely reduced compared to those in the wild type. This transcriptional gene silencing (TGS) was observed for transgenes under control of either the constitutive *CaMV 35S* promoter or the cold-inducible *RD29A* promoter. Bisulfite sequencing analysis revealed that both the transgene and endogenous *RD29A* promoter regions were hypermethylated at CG and non-CG contexts in the *rpt2a* mutant. Moreover, the TGS of transgenes driven by the *CaMV 35S* promoters was released by treatment with the DNA methylation inhibitor 5-aza-2′-deoxycytidine, but not by application of the inhibitor of histone deacetylase Trichostatin A. Genetic crosses with the DNA methyltransferase *met1* single or *drm1drm2cmt3* triple mutants also resulted in a release of *CaMV 35S* transgene TGS in the *rpt2a* mutant background. Increased methylation was also found at transposon sequences, suggesting that the 19S proteasome containing AtRPT2a negatively regulates TGS at transgenes and at specific endogenous genes through DNA methylation.

## Introduction

The 26S proteasome is an ATP-dependent proteinase complex that is responsible for regulated proteolysis of polyubiquitinated proteins in eukaryotic cells and is essential for the development of plants [1], [2]. The 26S proteasome is assembled from two particles: the 20S core particle (20S CP) and the 19S regulatory particle (19S RP). Proteolytic activities reside within the central chamber of the 20S CP, which is a hollow cylinder composed of four stacked rings [3], [4]. The 19S RP binds to one or both ends of the 20S CP and sits directly over the ring pore. The 19S RP recognizes polyubiquitinated proteins and is responsible for their ATP-dependent unfolding and threading through a narrow channel into the 20S CP [5]. The 19S RP is composed of two subcomplexes as follows: a base containing six related AAA-ATPases (designated RPT1–6 for regulatory particle triple-A ATPases) and three non-ATPase subunits (designated RPN1, RPN2, and RPN10, for regulatory particle non-triple-A ATPases), and a lid that contains at least 12 additional RPN subunits (RPN1–3 and −5–13). In plants, most genes encoding 19S RP subunits are duplicated. Such subunit duplication would lead to an increase in not only subunit redundancy but also subunit function. However, the functions of only some of the plant 19S RP subunits are known. RPT2 is essential for the channel opening of the α-ring of the 20S CP in yeast and mammals by its conserved C-terminal motif [6], [7]. The Arabidopsis genome contains two genes, *AtRPT2a* and *AtRPT2b* that are paralog RPT2 subunits with a difference of only four amino acids in the protein sequence. We have recently discovered that the *rpt2a* mutant shows a specific phenotype of enlarged leaves caused by increased cell size correlated with extended endoreduplication, whereas the *rpt2b* mutant did not show any morphological difference compared with the wild type [8].

DNA methylation is an important epigenetic mark for transcriptional gene silencing including genomic imprinting and repression of transposable elements in plants, vertebrates and some fungi [9], [10]. In general, cytosine methylation is found in both CG and non-CG (CHG and CHH where H is A, T or C) contexts in plants. In the model plant *Arabidopsis thaliana*, at least three methylation pathways exist and each is associated with a specific methyltransferase. DNA methylation is often considered a stable epigenetic mark, but active demethylation has been observed in both plants and animals and demethylases play an important role in protecting plant genes from potentially deleterious methylation [11]. In Arabidopsis, DNA glycosylases of the DEMETER (DME) family are responsible for removing methylcytosines from DNA. REPRESSOR OF SILENCING1 (ROS1), a DME homolog, is required for demethylating a transgene promoter and some endogenous genes, and for regulating their gene expression [12]. Plants maintain appropriate gene expressions and genomic stability with the coordination of methylases and demethylases.

During subsequent analysis of RPT2a by stable transformation with various expression constructs, many of these constructs showed gene silencing in the *rpt2a* mutant background. Here, we demonstrate a novel function of RPT2a for the specific regulation of gene silencing that involves DNA methylation in transgenes and some specific endogenous genes.

## Results and Discussion

### The *rpt2a* mutant showed transcriptional gene silencing

**Figure 1:**
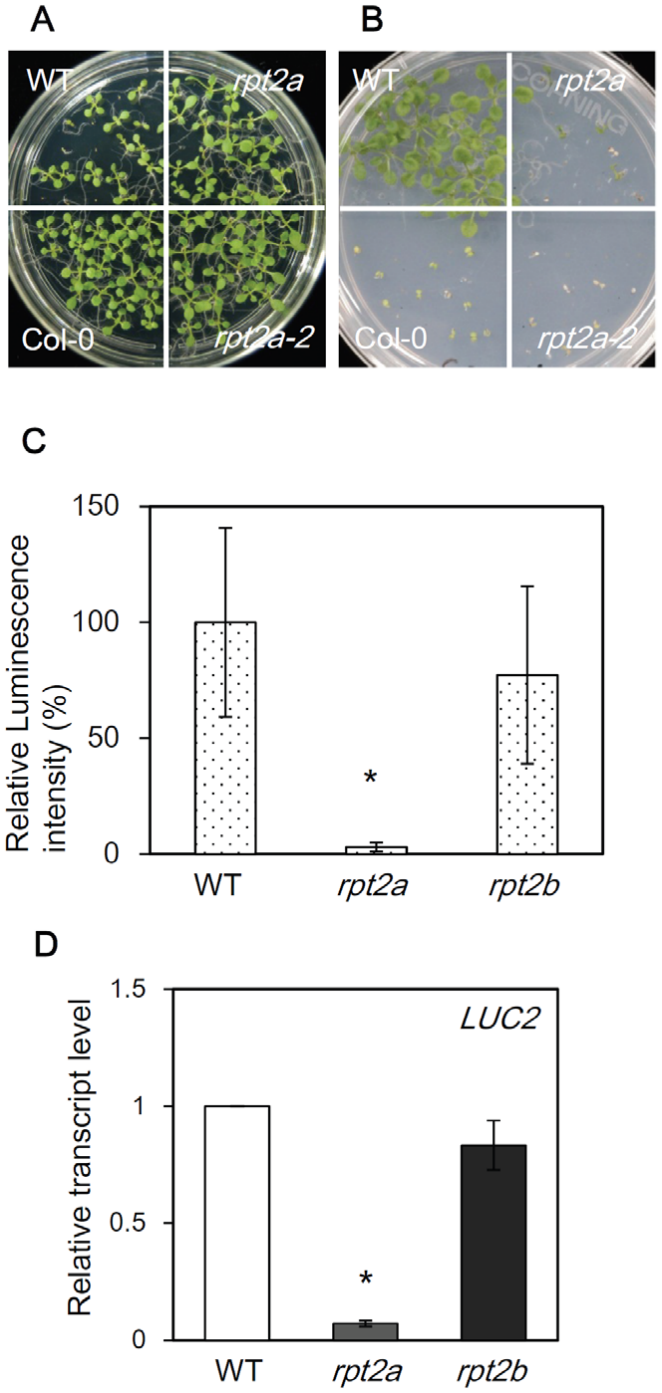
The *rpt2a* mutant shows transcriptional gene silencing. (A) 35S::HPT in the Col-0 (WT) and *rpt2a-2* mutant on MS medium. Col-0 plants without any transgene indicate “Col-0”. (B) 35S::HPT in the WT and *rpt2a-2* mutant on MS medium containing 50 µM hygromycin. (C) Relative luminescence intensity of 35S::LUC2 in WT, *rpt2a-2* and *rpt2b-1* mutants. 35S::LUC2 in WT is set as 100%. *t-test P<0.05, error bar = S.D., n = 20. (D) Quantification of LUC2 gene expression in 35S::LUC2 in WT, *rpt2a-2* and *rpt2b-1* mutants. 35S::LUC2 in WT is set as 1. Values are the averages of the three experiments, and the level of *18S rRNA* was used as an internal control.

We found that the *rpt2a-2* mutant displayed a phenotype of repressed transgene expression during construction of transgenic plants for investigating RPT2a function. As an example of this, introduction of the hygromycin B phosphotransferase gene (HPT) driven by the constitutive *CaMV 35S* promoter into Col-0 wild-type plants conferred plant survival on MS medium containing hygromycin in Col-0 background. In contrast, the transgene became inactive in *rpt2a-2* and transgenic plants showed sensitivity to hygromycin-containing media (Figure 1A and 1B). In order to examine the function of AtRPT2a in transgene silencing quantitatively, luciferase2 (LUC2) overexpressing plants were produced in *rpt2a-2* and *rpt2b-1* mutant backgrounds by crossing with transgenic wild-type. We confirmed that the transgenic plants expressing LUC2 under the *CaMV* 35S promoter have a single copy T-DNA inserted in the euchromatin region (Figure S2A, 2C, 2E). Although AtRPT2a and AtRPT2b share an almost identical amino acid sequence, only the *rpt2a-2* mutant showed one-tenth the luminescence of the WT, and the *rpt2b-1* mutant showed the same level of luminescence as WT (Figure 1C). An identical result was obtained with the *rpt2a-1* allele (Figure S1). This result suggests that RPT2a may regulate the expression of transgenes.

To determine whether repression of the transgene in the *rpt2a* mutant was regulated at the transcriptional or post-translational level, PT-PCR was used to examine the accumulation of *LUC2* transcripts. Accumulation of *LUC2* transcripts was dramatically decreased in the *rpt2a-2* mutant compared to that in the WT (Figure 1D). Consistent with that observed for luciferase activity, *LUC2* transcript accumulation in the *rpt2b-1* mutant was not different to that in WT (Figure 1D). These results suggest that the luciferase gene was repressed at the transcriptional level in the *rpt2a* mutant.

### The DNA methylation inhibitor released transcriptional gene silencing in the *rpt2a* mutant

**Figure 2:**
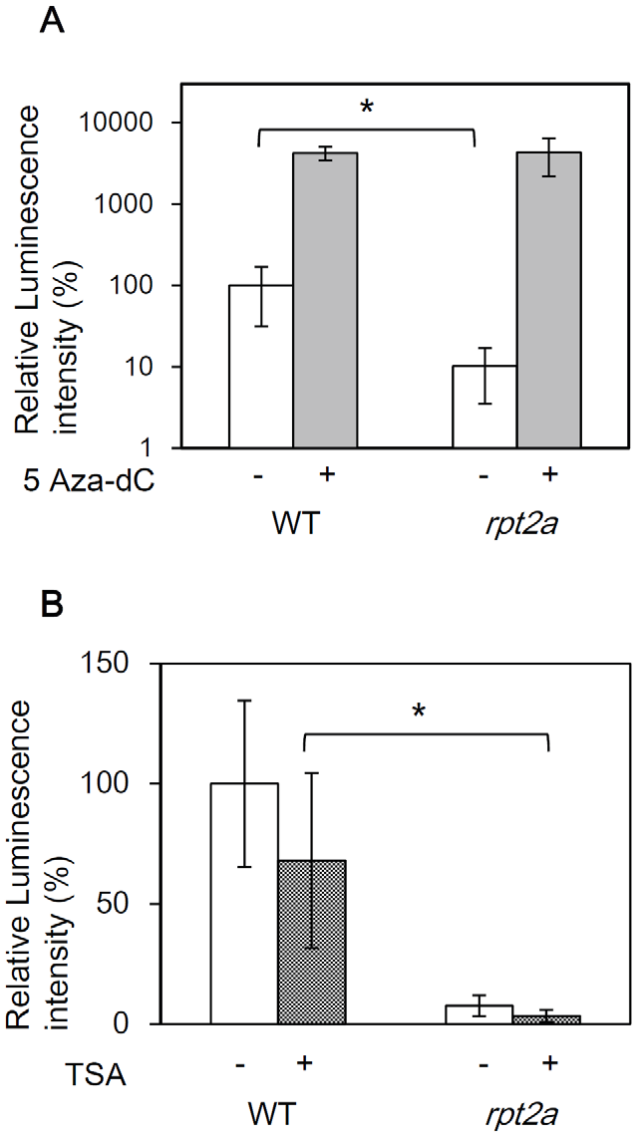
5-aza-2′-deoxycytidine (5Aza-dC) treatment releases gene silencing in the *rpt2a* mutant. (A) Relative luminescence intensity of WT and the *rpt2a-2* mutant treated with 50 µM 5-aza-2′-deoxycytidine (5Aza-dC). Seedlings grown in MS medium for 2 weeks are transferred to MS liquid medium containing 50 µM 5Aza-dC for one week. 35S::LUC2 in WT without 5Aza-dC treatment is set as 100%. *t-test P<0.05, error bar = S.D., n = 15. (B) Relative luminescence intensity of WT and the *rpt2a-2* mutant treated with 0.1 µM TrichostatinA (TSA). Seedlings grown in MS medium for 2 weeks are transferred to MS liquid medium containing 0.1 µM TSA for one week. 35S::LUC2 in WT without TSA treatment is set as 100%. *t-test P<0.05, error bar = S.D., n = 15.

Transcriptional gene silencing (TGS) is often associated with DNA methylation and histone modification. To examine whether such epigenetic changes were involved in the repression observed in the *rpt2a* mutant, we tested an inhibitor of cytosine methylation, 5-aza-2′-deoxycytidine (5Aza-dC) and an inhibitor of histone deacetylase, TrichostatinA (TSA). Following treatment with 5Aza-dC, gene silencing in the *rpt2a-2* mutant was released at the same level as that of 5Aza-dC treated WT (Figure 2A). On the other hand, treatment with TSA did not cause a change in luciferase activity in the *rpt2a* mutant (Figure 2B). From the experiments using the genes that have been reported to be transcriptionally increased by TSA treatment [13], [14], we confirmed that the TSA treatment is effective (Figure S3). Although these results could not deny the possibility that histone modification, except acetylation, is involved in gene silencing in *rpt2a* mutant, these results suggest that DNA hypermethylation is correlated with gene silencing in the *rpt2a* mutant.

### The mutation of DNA methyltransferase released gene silencing in the *rpt2a* mutant

**Figure 3:**
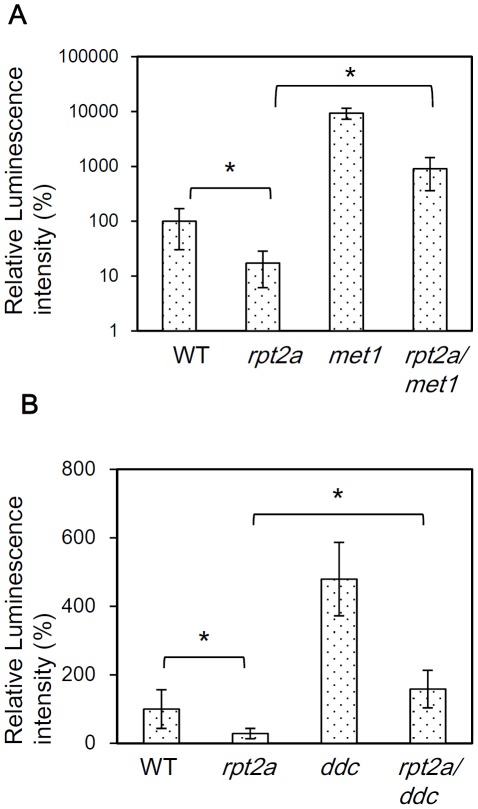
DNA methyltransferase mutants release gene silencing in the *rpt2a* mutant. (A) Relative luminescence intensity of WT, *rpt2a-2*, *met1-1* and *rpt2a-2met1-1* double mutants. 35S::LUC2 in WT is set as 100%. *t-test P<0.05, error bar = S.D., n = 10. (B) Relative luminescence intensity of WT, *rpt2a-2*, *drm1 drm2 cmt3* triple mutant (*ddc*) and *rpt2a-2 drm1 drm2 cmt3* quadruple mutant (*rpt2a/ddc*). 35S::LUC2 in WT is set as 100%. *t-test P<0.05, error bar = S.D., n = 20.

Three classes of DNA methyltransferases: MET1, CMT3 and DRM1/2, regulate DNA methylation in Arabidopsis thaliana. MET1 maintains CG methylation, while DRM1/2 and CMT3 are responsible for methylation at non-CG sites [15]. The above data show that DNA methylation was involved in transgene silencing in the *rpt2a* mutant (Figure 2A). To further investigate the relationship between gene silencing in the *rpt2a* mutant and DNA methylation, we next examined luciferase activity in an *rpt2a-2 met1-1* double mutant. In addition, DRM1, DRM2 and CMT3 are reported to have a redundant function [16] and thus, we made a *rpt2a-2 drm1 drm2 cmt3* quadruple mutant and checked the luciferase activity in this background. *LUC* activity was found to be much higher in the *rpt2a-2 met1-1* double mutant compared to that of the single *rpt2a-2* mutant although this was still lower than that observed in the *met1* single mutant (Figure 3A). This result suggests a partial release of gene silencing by *met1* in the *rpt2a* mutant.

*LUC* activity in the *rpt2a-2 drm1 drm2 cmt3* quadruple mutant was also higher than that in the single *rpt2a-2* mutant, whereas that of the *drm1 drm2 cmt3* triple mutant was higher than that in the quadruple mutant (Figure 3B). This result suggests a partial release of gene silencing in the *rpt2a* mutant by the *drm1drm2cmt3* triple mutant. These results are consistent with gene silencing in the *rpt2a* mutant caused by both CG and non-CG hypermethylation. The *rpt2a-2 met1* double mutant showed much higher *LUC* activity than that of the *rpt2a-2 drm1 drm2 cmt3* quadruple mutant, due to a greater influence of CG methylation on the *as-1* regulatory element within the *CaMV 35S* promoter [17].

### The *rpt2a* mutation leads to DNA hypermethylation in the promoter of the silenced loci

**Figure 4:**
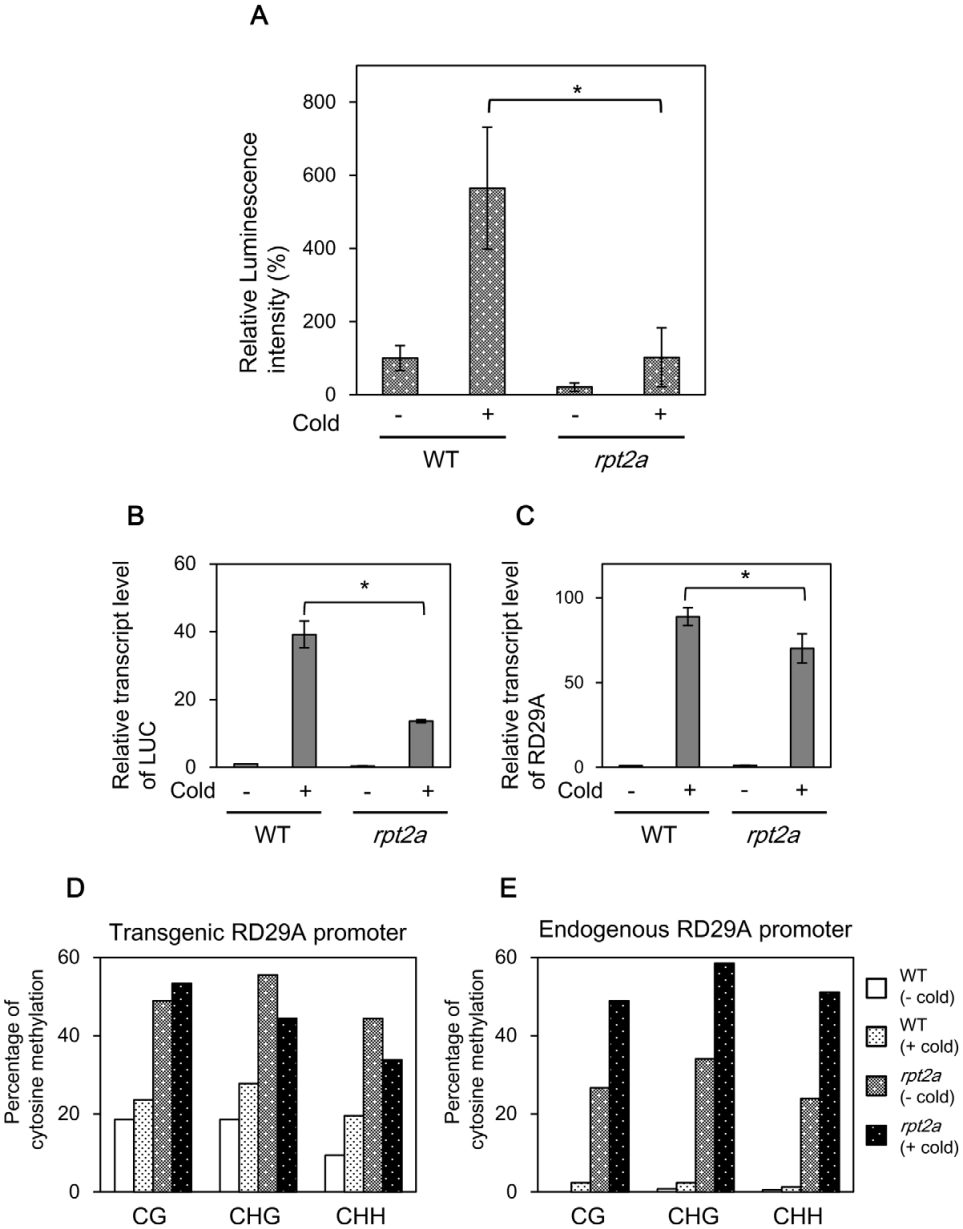
Gene silencing in the *rpt2a* mutant is correlated with DNA hypermethylation. (A) Relative luminescence intensity of RD29A::LUC in WT and *rpt2a-2*. Plants are untreated or treated with cold (4°C) for 12 hours. (B) Quantification of *LUC* gene expression in RD29A::LUC in WT and *rpt2a-2*. Expression levels are relative to that of untreated WT plants. Values are the average of three experiments, and the level of *18S rRNA* was used as an internal control. (C) Quantification of *RD29A* gene expression in RD29A::LUC in WT and *rpt2a-2*. Expression levels are relative to that of untreated WT plants. Values are the averages of three experiments, and the level of *18S rRNA* is used as an internal control. (D) Mean levels of DNA methylation in different cytosine context at the exogenous *RD29A* promoter in WT and the *rpt2a-2* mutant. Frequencies of methylcytosine at CG, CHG and CHH sites are indicated. Twenty clones are sequenced for each sample. (E) Mean levels of DNA methylation in different cytosine contexts at the endogenous *RD29A* promoter in WT and the *rpt2a-2* mutant.

To confirm that the DNA methylation is increased in the *rpt2a* mutant, we checked the methylation level of the *rpt2a* mutant by bisulfite sequencing. Methylation levels in the *CaMV 35S* promoter increased in the *rpt2a* mutant compared to that in WT. However, the *CaMV 35S* promoter was also highly methylated in WT, consistent with the results of 5Aza-dC treatment and genetic analysis of DNA methyltransferases, and the differences in DNA methylation level between WT and the *rpt2a* mutant were obscure. We therefore placed the *LUC* gene under control of the cold- and drought-responsive *RD29A* promoter [18]. We confirmed that transgenic plants expressing LUC under the *RD29A* promoter have a single copy T-DNA inserted in the euchromatin region (Figure S2B, 2D, 2F). Luminescence was induced in WT containing *RD29A::LUC* when plants were treated with cold stress for 12 hours. On the other hand, luminescence was repressed in *rpt2a-2* with and without low temperature treatment (Figure 4A). We confirmed that cold treatment induced transcript accumulation of *LUC* and endogenous *RD29A* in WT, whereas these transcripts were repressed in *rpt2a-2* upon the cold treatment (Figure 4B, 4C). In contrast, gene expression of other cold-responsive genes *COR15A* and *DREB1* were induced in *rpt2a-2* (Figure S4). These results reveal that the *rpt2a* mutant shows gene silencing of the genes under control of both the *RD29A* and *CaMV 35S* promoter, suggesting that TGS in the *rpt2a* mutant is independent of promoter sequences. DNA methylation levels of exogenous and endogenous *RD29A* promoters were next investigated. Compared to that in WT, the methylation level of the exogenous *RD29A* promoter increased and broadened in the *rpt2a* mutant both before and after cold treatment (Figure 4D, S5). This result may imply that demethylation of the *RD29A* promoter was abnormal in the *rpt2a* mutant. The endogenous *RD29A* promoter contained a very low level of DNA methylation in WT before and after cold treatment. On the other hand, the methylation level of the endogenous *RD29A* promoter increased in the *rpt2a* mutant both before and after cold treatment (Figure 4E). The *RD29A* promoter contains a *cis*-acting dehydration-responsive element (DRE) involved in the induction by exposure to low temperature [18]. After cold treatment, the methylation level of DRE in endogenous *RD29A* promoter greatly increased in *rpt2a-2* suggesting that DNA methylation of DRE represses the transcription of endogenous *RD29A* genes in *rpt2a-2* (Figure S6). These results are consistent with TGS of transgenes in the *rpt2a* mutant caused by increased DNA methylation in the promoter region. Interestingly, cold treatment also induced a slight increase of DNA methylation in the Col-0 background (Figure S7). This result may indicate that a rapid increase of transcription induces DNA methylation for marking of activated genes and for monitoring of genome stability.

### The transposable element is highly methylated in the *rpt2a* mutant

**Figure 5:**
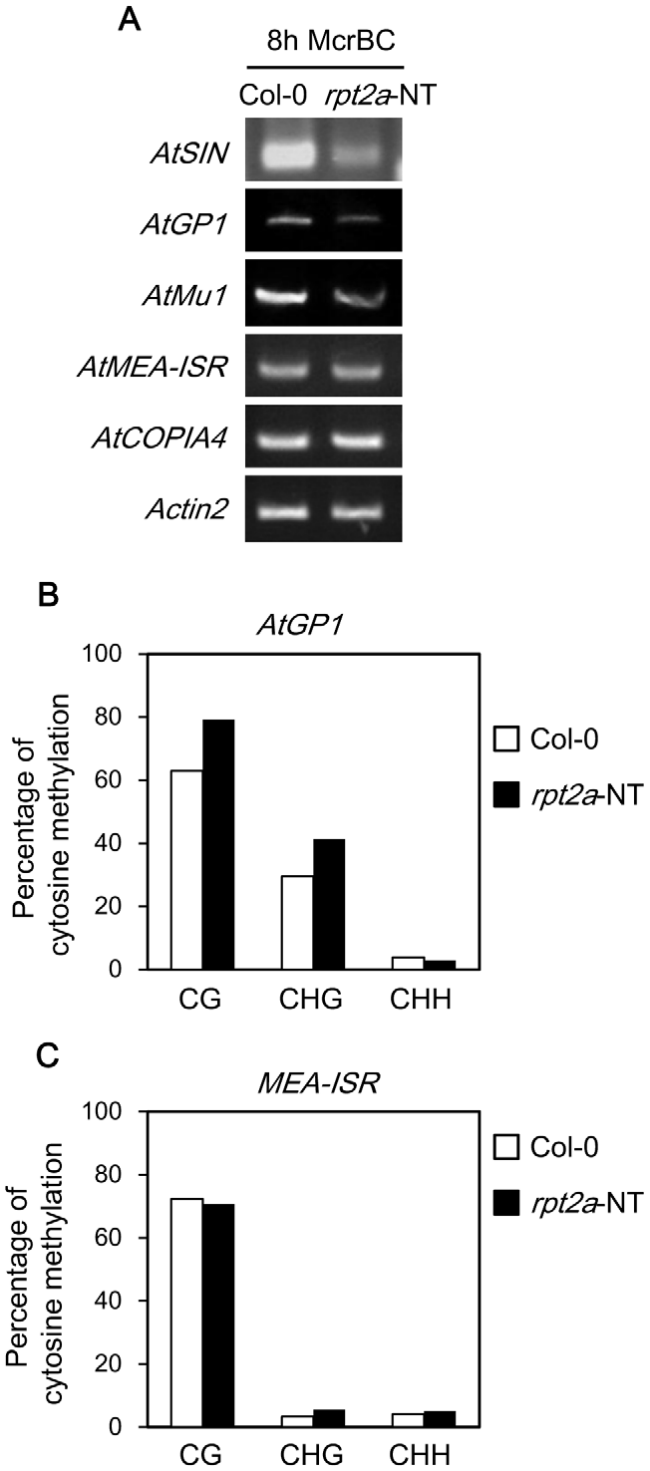
DNA methylation level of transposons is increased in the *rpt2a* mutant. (A) McrBC PCR of transposons at Col-0 and *rpt2a-2* (no transgene: *rpt2a*-NT). McrBC-digested genomic DNA is amplified by PCR with primers for the indicated transposons. Input DNA was normalized for each genotype with *Actin2*. (B) Mean levels of DNA methylation in different cytosine context at the *AtGP1* in Col-0 and *rpt2a-2* (no transgene). (C) Mean levels of DNA methylation in different cytosine context at the *MEA-ISR* in Col-0 and *rpt2a-2* (no transgene).

We have shown that the DNA methylation level of the promoter site of transgenes increased in the *rpt2a* mutant. We next tested the hypothesis that endogenous genes were also hypermethylated in the *rpt2a* mutant. The *Arabidopsis* genome contains silenced transposable elements. We tested methylation levels at several transposons by methylation-sensitive PCR with McrBC, which preferentially cuts methylated DNA [19]. Higher levels of methylation result in increased McrBC digestion and consequently reduced amplification products. Compared with Col-0, methylation levels of *AtSINE1* and *AtGP1* increased in the *rpt2a-2* (Figure 5A). On the other hand, the methylation level of *AtCOPIA4* and *AtMEA-ISR* were not different from Col-0. The methylation levels of *AtGP1* and *AtMEA-ISR* were examined further by bisulfite sequencing analysis. We confirmed that the methylation level of *AtGP1* increased in *rpt2a-2*, but that of *AtMEA-ISR* was not significantly different from Col-0 (Figure 5B, 5C, S8). This result shows that DNA methylations increased in the *rpt2a* mutant in specific genome loci, but not in the whole genome.

We show here that the loss of At*RPT2a* function results in TGS and an increase in the DNA methylation of promoter sequences of transgenes. Transposons also showed hypermethylation in the *rpt2a* mutant. These observations suggest that AtRPT2a, a 19S proteasome subunit protein, is required for the negative regulation of DNA methylation at transgenes and specific genome loci. This is the first report that the proteasome has the potential of regulating DNA methylation.

Genome-wide methylation analysis has shown that about 30% of genes are methylated in Arabidopsis [20] and this DNA methylation status is dynamically regulated by DNA methylation and demethylation reactions [11]. We showed that hypermethylation in *rpt2a-2* involved all three methyltransferases. There was no difference in the expression level of these genes for the DNA methyltransferase between Col-0 and *rpt2a-2* (Figure S9). PEST motif prediction (http://mobyle.pasteur.fr/cgi-bin/portal.py?#forms::epestfind) showed that MET1, CMT3 and DRM1/2 all contain PEST motifs suggesting that these DNA methyltransferases can be degraded by the 26S proteasome. Taken together, hypermethylation in the *rpt2a* mutant is thought to be due to the accumulation of DNA methyltransferase. This hypothesis is supported by a report that, in mammals, Dnmt1 (the homolog of MET1) is degraded by proteasomes upon treatment with a DNA methylation inhibitor [21]. However, increased methylation in *rpt2a* was observed at specific loci rather than globally, indicating that the accumulation of DNA methyltransferases alone was not responsible for hypermethylation in the *rpt2a* mutant.

ROS1 and ROS3 are required for demethylation [12], [22]. Mutations in ROS1 cause hypermethylation of the *RD29A* promoter, leading to silencing of the transgene and its homologous endogenous gene. ROS1 is also required to suppress DNA methylation in a number of other endogenous genomic loci including many transposons [23], [24]. Since we showed that the *rpt2a* mutant shows a similar phenotype to the *ros1* mutant, a hypothesis is presented that AtRPT2a could function with ROS1 and ROS3 in a demethylation pathway. Unfortunately, we have not excluded the relationship between the RPT2a and ROS pathway in this report.

Recent works raise an alternative hypothesis for the function of AtRPT2a. The 19S RP has been shown to be required for methylation of histone H3 lysine 4 (H3K4) in yeast and mammals [25], [26]. In Arabidopsis, genome-wide analysis showed that DNA methylation and H3K4 di- and tri-methylation are mutually exclusive [27]. Although TSA treatment did not release gene silencing in the *rpt2a* mutant in this report, these observations raise the possibility that an increase of DNA methylation in the *rpt2a* mutant may be caused by a decrease of H3K4 methylation and expansion of DNA methylation. We could not totally rule out the possibility that the proteasome indirectly controls DNA methylation since the ubiquitin/26S proteasome pathway regulates many biological phenomena. Further studies are required to determine the causes of hypermethylation in the *rpt2a* mutant.

Epigenetic modification is an important mechanism for adaption of gene expression to development and environmental status [28]. In addition, the Ub/proteasome system plays a crucial role in the response of hormone signaling and environmental stress [2], [29]. We have demonstrated that the RPT2a subunit is required for the regulation of DNA methylation, suggesting that the proteasome participates in epigenetic modification for proper gene expression depending on the environmental status.

## Materials and Methods

### Plant materials

For germination of *Arabidopsis thaliana* (ecotype Columbia-0) wild type and mutants, seeds were surface-sterilized and placed on Murashige and Skoog (MS) medium supplemented with 2% sucrose (Germination inducible medium: GIM). After cold treatment for 2 days to synchronize germination, seeds were transferred to 22°C and 50% relative humidity under a 16/8 h light/dark cycle (this time point indicates 0 days after sowing: DAS). The seeds of the *met1-1* mutant were provided by Dr. Robert A. Martienssen (Cold Spring Harbor Laboratory). Seeds of the *rpt2a-1*, *rpt2a-2*, *rpt2b-1*, and *drm1 drm2 cmt3 triple* mutants were obtained from the ABRC (The Arabidopsis Biological Resource Center, Ohio State University, Columbus, OH, USA; stock number: SALK_130019, SALK_005596, SALK_043450, and CS16384 respectively). Sequences bordering the T-DNA insertion were determined using primer pairs listed in Table S1. 35S::HPT plants were obtained by transformation of the wild-type Arabidopsis plants of the Columbia-0 ecotype with T-DNA composed of a HPT gene conferring resistance to hygromycin driven by the 35S promoter of the cauliflower mosaic virus. 35S::LUC2 plants were obtained by transformation of Columbia-0 with a destination vector p7-LUC2. Mutants were crossed to each transgenic line, and F3 progenies homozygous for transgenes and/or the mutations were used for experiments. RD29A::LUC plants were obtained by transformation of Columbia-0 with a destination vector pGWB35 containing genomic fragments of the promoter region of RD29A (824-bp upstream of the ATG). Mutants were crossed to each transgenic line, and F3 progenies homozygous for the transgenes and/or the mutations were used for experiments.

For 5-aza-2′-deoxycytidine (5Aza-dC) treatment and TrichostatinA (TSA) treatment, seedlings grown for one week were transferred to MS liquid medium containing 50 µM 5Aza-dC (Wako) or to MS liquid medium containing 0.1 µM TSA (Wako).

### Luciferase activity

Five millimeter diameter leaf sections were floated on 50 µl of Pikkagene cell lysis buffer (TOYO B-Net. CO., LTD) containing 20 µl of 0.1 µM D-Luciferine potassium salt and incubated for 30 min. Samples were measured using a Luminescenceor JNR II (Atto).

### Transcript level analysis

Total RNA was extracted by the guanidine thiocyanate method [30]. Total RNA (0.6 µg RNA) was used as a template for first strand cDNA synthesis with ReverTraAce -α-® reverse transcriptase (TOYOBO, Osaka, Japan). First strand cDNA (0.7 µl) was then assayed for gene-specific DNA fragments using the primer pairs listed in Table S1. PCR amplification was performed in the optimum cycles with each gene using the Taq DNA polymerase (New England BioLabs® Japan inc, Tokyo, Japan). Amplified fragments were separated on 1.2% (w/v) agarose gels and visualized by ethidium bromide staining. Real-time PCR was performed with the Power SYBR Green PCR Master Mix (Applied Biosystems) on an Applied Biosystems 7300 Real-Time PCR system (Applied Biosystems). Relative quantitation of gene expression is based on the comparative CT method (User Bulletin No. 2: ABI PRISM 7700 Sequence Detection System, 1997) using *18S rRNA* as a reference gene. The following PCR program was used: 2 min at 50°C; 10 min at 95°C; 40 cycles of 15 sec at 95°C, and 1 min at 60°C. Two biological and three technical replicates were performed. The sequences of the primers used are specified in Table S1.

### Bisulfite sequencing

For analysis of DNA methylation by bisulfite sequencing, DNA was isolated from the first leaves of 3-week-old plants of WT and mutants using a Nucleon PhytoPure DNA extraction kit (GE healthcare). The protocol of bisulfite treatment in this study is based on the methods of Kanazawa et al., 2007 [31]. DNA was cleaved with the restriction enzyme EcoRI, extracted with phenol/chloroform, and precipitated by ethanol. The cleaved DNA was alkali denatured in 0.3 M NaOH at 37°C for 40 min. Denatured DNA was incubated in a total volume of 600 µM with freshly prepared 5.9 M urea/3.35 M sodium bisulfite/0.5 mM hydroquinone pH 5.0, at 60°C for 36 h under mineral oil. A Quick PCR purification kit (Qiagen) then recovered the DNA. NaOH was added to the DNA solution to a concentration of 0.3 M and then incubated at 37°C for 30 min. Glycogen and ammonium acetate were added to the solution to final concentrations of 0.16 mg/ml and 2.64 M, respectively. DNA was then precipitated with ethanol and dissolved in 10 µl of TE (pH 8.0). Two rounds of PCR were carried out using 1 µl of bisulfite-treated DNAs as a template. Primers for the RD29A promoter and transposons were modified based on the methods of Zheng et al. (2008) and Gao et al. (2010) [22], [32]. To amplify the exogenous RD29A promoter, primers pRD29A nested and pRD29A transR1 were used for the first round PCR, and primers pRD29A converted and pRD29A transR2 were used for the second round PCR. To amplify the endogenous RD29A promoter, primers pRD29A nested and pRD29A endoR1 were used for the first round PCR, and primers pRD29A converted and pRD29A endoR2 were used for the second round PCR. The PCR products were cloned into a pCR2.1 vector (Invitrogen) and 20 clones per one plant were subjected to sequence analysis.

### McrBC PCR

McrBC PCR was performed on genomic DNA that was extracted from 3-week-old rosette leaves from 5 plants grown under identical conditions as described above. 250 ng genomic DNA was digested with McrBC for 3 hours and assayed using the PCR primers listed in Table S1.

## Supporting Information

Figure S1 Relative luminescence intensity of 35S::LUC2 in WT and *rpt2a-1*. *t-test P<0.05, error bar = S.D., n = 25.
(TIF)

Figure S2 Analysis of T-DNA insertion site. (A) Southern blot analysis of Col-0, 35S::LUC2 in WT and in *rpt2a-2* genomic DNA with the LUC2 as a probe. (B) Southern blot analysis of Col-0, RD29A::LUC in WT and in *rpt2a-2* genomic DNA with the LUC as a probe (Methods S1). (C) 35S::LUC2 T-DNA insertion site in *At5g58580*. (D) RD29A::LUC T-DNA insertion site in *At3g11860*. (E) Insertion check of 35S::LUC2 by PCR. (F) Insertion check of RD29A::LUC.
(TIF)

Figure S3 RT-PCR analysis of TSA treated plants: *ABI3*, *At3g29650* and *18S rRNA* (control).
(TIF)

Figure S4 (A) RT-PCR analysis of cold inducible genes: *COR15A*, *DREB1B* and *18S rRNA* (control). (B) Quantification of *RD29A* gene expression in Col-0 and *rpt2a-2* (no transgene: *rpt2a*-NT). Expression levels are relative to that of untreated WT plants. Values are the averages of three experiments, and the level of *18S rRNA* is used as an internal control.
(TIF)

Figure S5 (A) Scheme of analyzed region in exogenous *RD29A* promoter. (B) Bisulfite sequencing of DNA methylation in the exogenous *RD29A* promoter site (from −346 bp to −51 bp upstream of the promoter). Upper graph shows methylation status in WT and the lower graph shows DNA methylation status in the *rpt2a-2* mutant. The height of the vertical lines shows the frequency of methylcytosine. Red, blue and green lines indicate frequencies of methylcytosine at CG, CHG and CHH sites, respectively. Red bars on the x-axis are DRE and DRE/CRT core sequences. Twenty clones are sequenced for each sample.
(TIF)

Figure S6 (A) Scheme of analyzed region in endogenous *RD29A* promoter. (B) Bisulfite sequencing of DNA methylation in the endogenous *RD29A* promoter site (from −327 bp to −32 bp upstream of promoter). The upper graph shows methylation status in WT and the lower graph shows DNA methylation status in the *rpt2a-2* mutant. The height of the vertical lines shows the frequency of methylcytosines. Red, blue and green lines indicate frequencies of methylcytosine at CG, CHG and CHH sites, respectively. Red bars on the x-axis are DRE and DRE/CRT core sequences. Twenty clones are sequenced for each sample.
(TIF)

Figure S7 (A) Bisulfite sequencing of DNA methylation in the endogenous *RD29A* promoter site (from −327 bp to −32 bp upstream of the promoter) in Col-0 and *rpt2a-2* (no transgene: *rpt2a*-NT). The upper graph shows methylation status in cold-untreated and treated Col-0, and the lower graph shows cold-untreated and treated *rpt2a-2* (no transgene). (B) Mean levels of DNA methylation in different cytosine context at the exogenous and endogenous *RD29A* promoter in Col-0 and *rpt2a-2* (no transgene). Red, blue and green lines indicate frequencies of methylcytosine at CG, CHG and CHH sites, respectively. Twenty clones are sequenced for each sample.
(TIF)

Figure S8 Bisulfite sequencing of DNA methylation in the *AtGP1* site in Col-0 and *rpt2a-2* (no transgene: *rpt2a*-NT).
(TIF)

Figure S9 RT-PCR analysis of DNA methyltransferase genes: *MET1*, *CMT3*, *DRM2* and *18S rRNA* (control).
(TIF)

Table S1 Primers used in this study.
(PDF)

Methods S1 Supplementary methods.
(PDF)
