# Supplementary figures and images for: Arabidopsis RPT2a, 19S Proteasome Subunit, Regulates Gene Silencing via DNA Methylation
Source: PLoS One. 2012 May 16;7(5):e37086. doi: 10.1371/journal.pone.0037086 (PMC3353898; doi:10.1371/journal.pone.0037086)

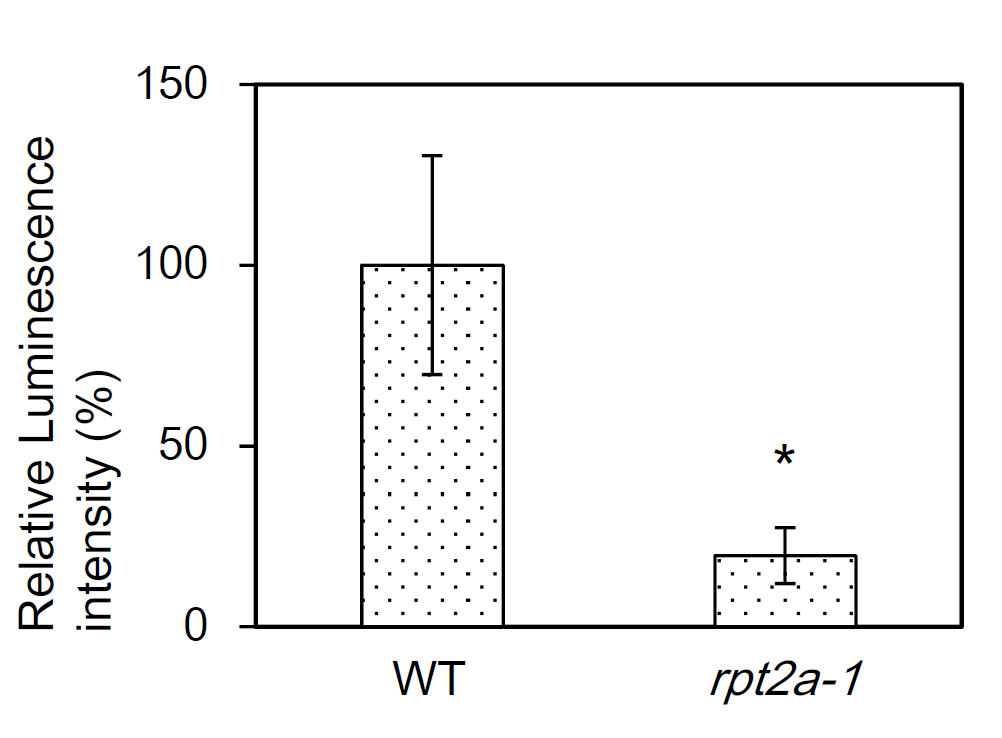

Supplement: Figure S1 — Relative luminescence intensity of 35S::LUC2 in WT and rpt2a-1. *t-test P<0.05, error bar = S.D., n = 25. (TIF) [file pone.0037086.s001.tif]

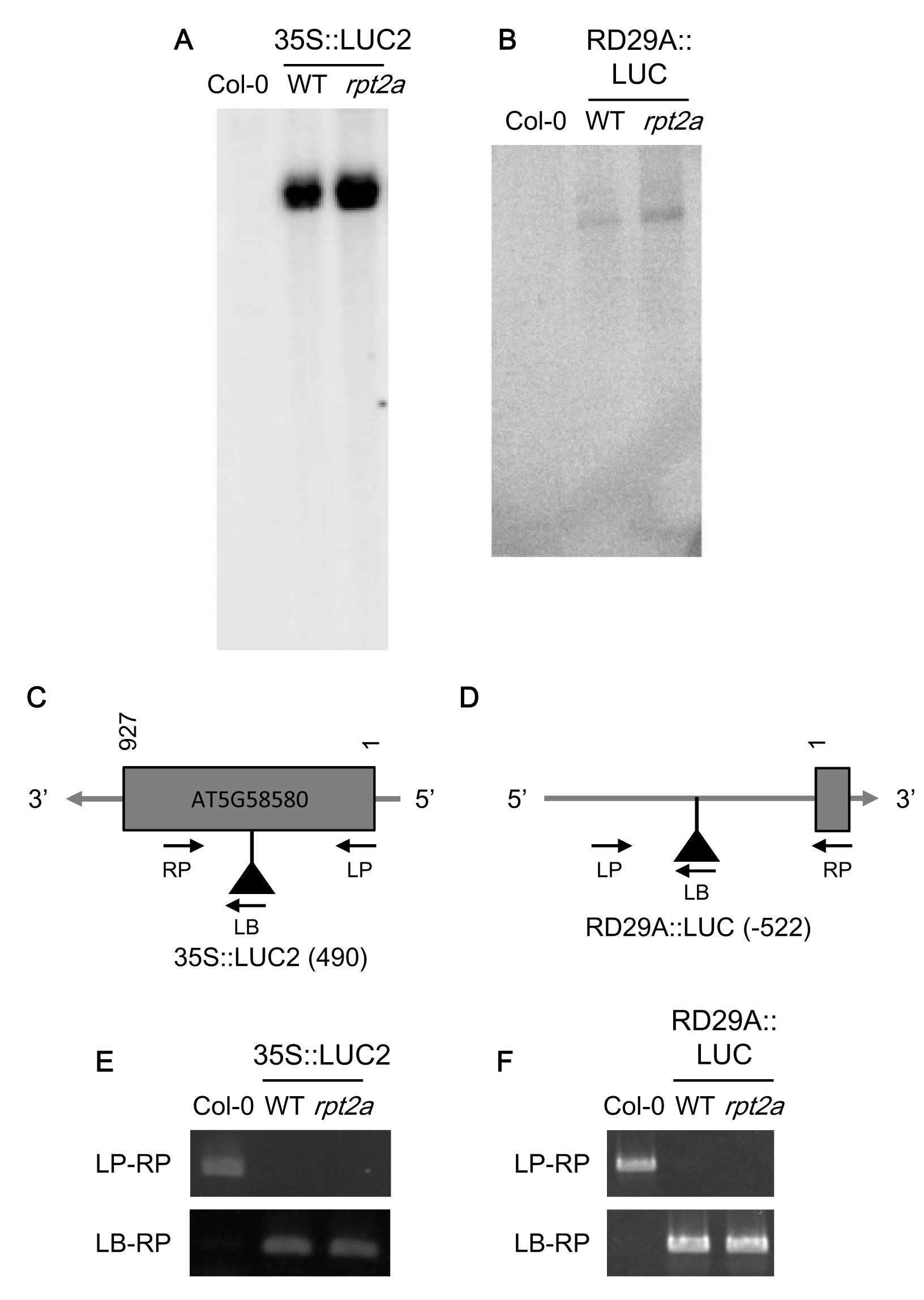

Supplement: Figure S2 — Analysis of T-DNA insertion site. (A) Southern blot analysis of Col-0, 35S::LUC2 in WT and in rpt2a-2 genomic DNA with the LUC2 as a probe. (B) Southern blot analysis of Col-0, RD29A::LUC in WT and in rpt2a-2 genomic DNA with the LUC as a probe (Methods S1). (C) 35S::LUC2 T-DNA insertion site in At5g58580. (D) RD29A::LUC T-DNA insertion site in At3g11860. (E) Insertion check of 35S::LUC2 by PCR. (F) Insertion check of RD29A::LUC. (TIF) [file pone.0037086.s002.tif]

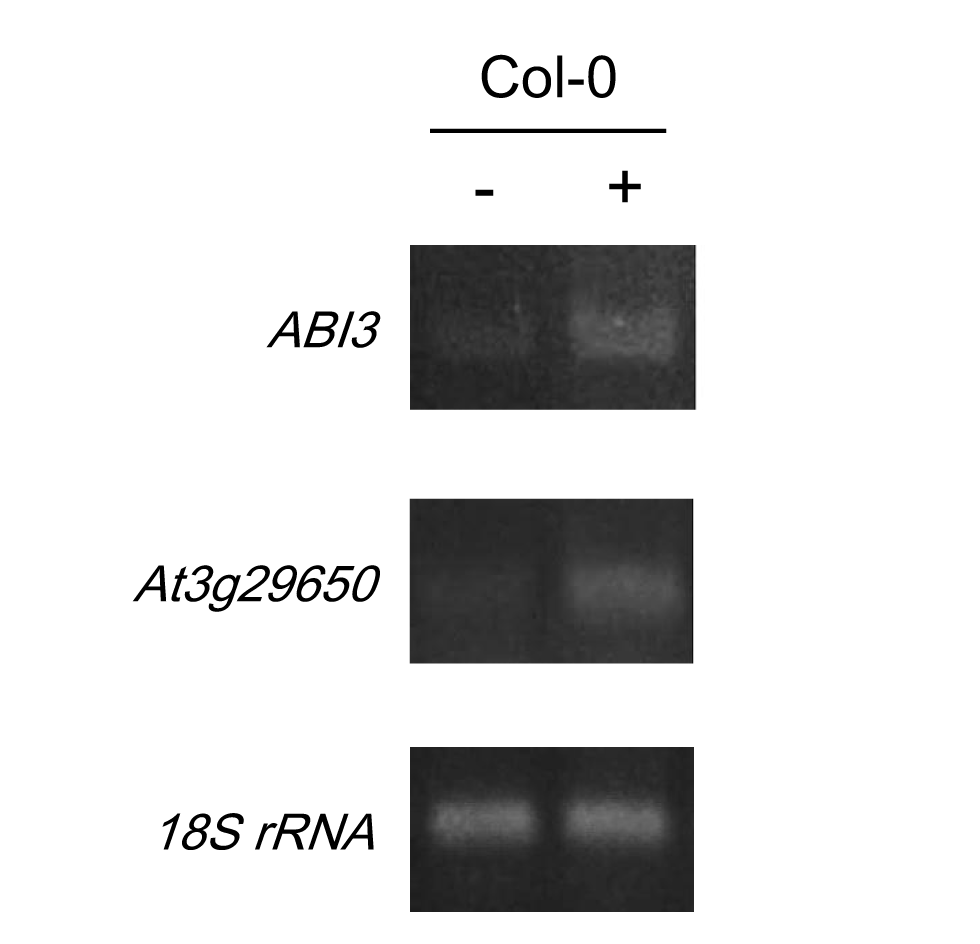

Supplement: Figure S3 — RT-PCR analysis of TSA treated plants: ABI3, At3g29650 and 18S rRNA (control). (TIF) [file pone.0037086.s003.tif]

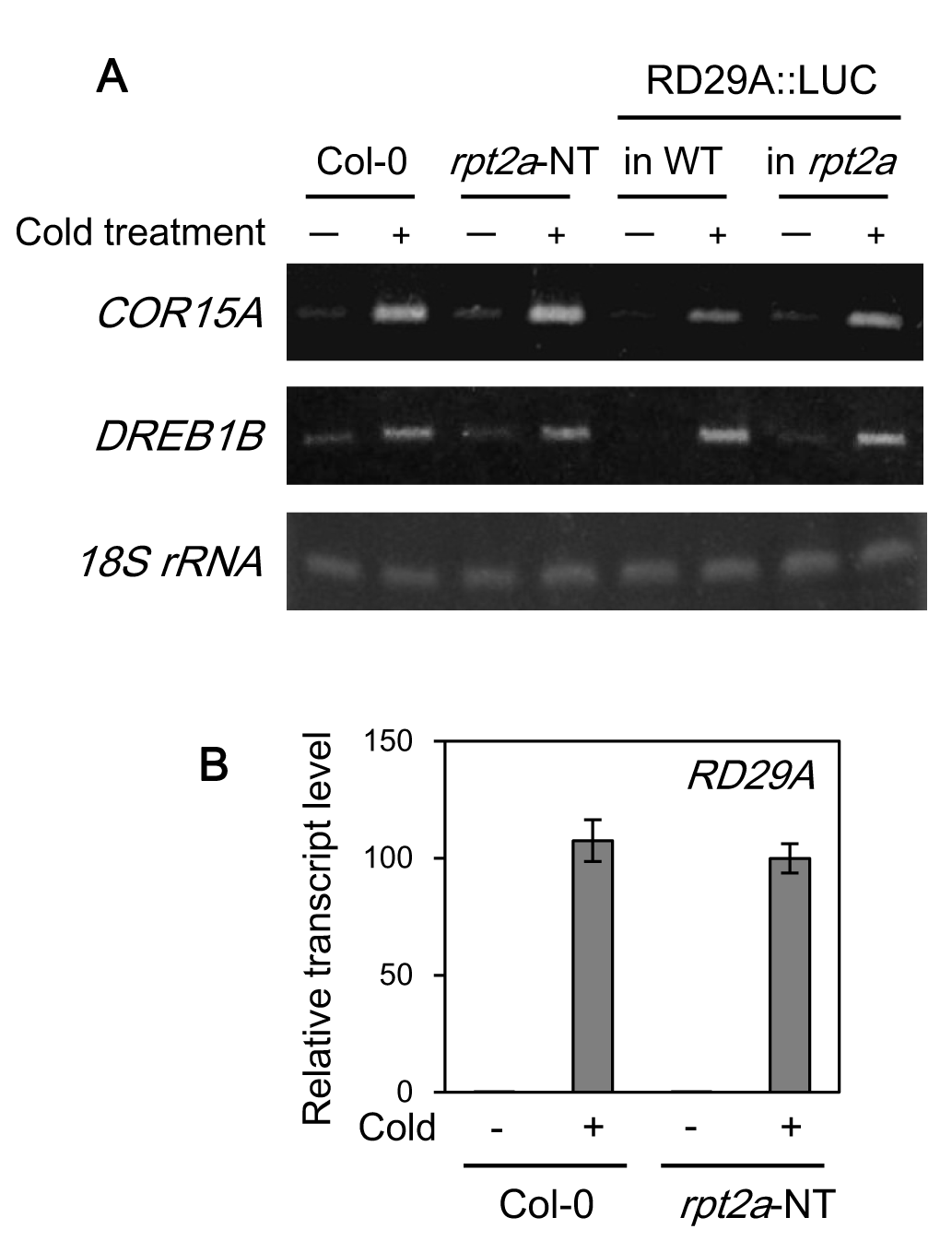

Supplement: Figure S4 — (A) RT-PCR analysis of cold inducible genes: COR15A, DREB1B and 18S rRNA (control). (B) Quantification of RD29A gene expression in Col-0 and rpt2a-2 (no transgene: rpt2a-NT). Expression levels are relative to that of untreated WT plants. Values are the averages of three experiments, and the level of 18S rRNA is used as an internal control. (TIF) [file pone.0037086.s004.tif]

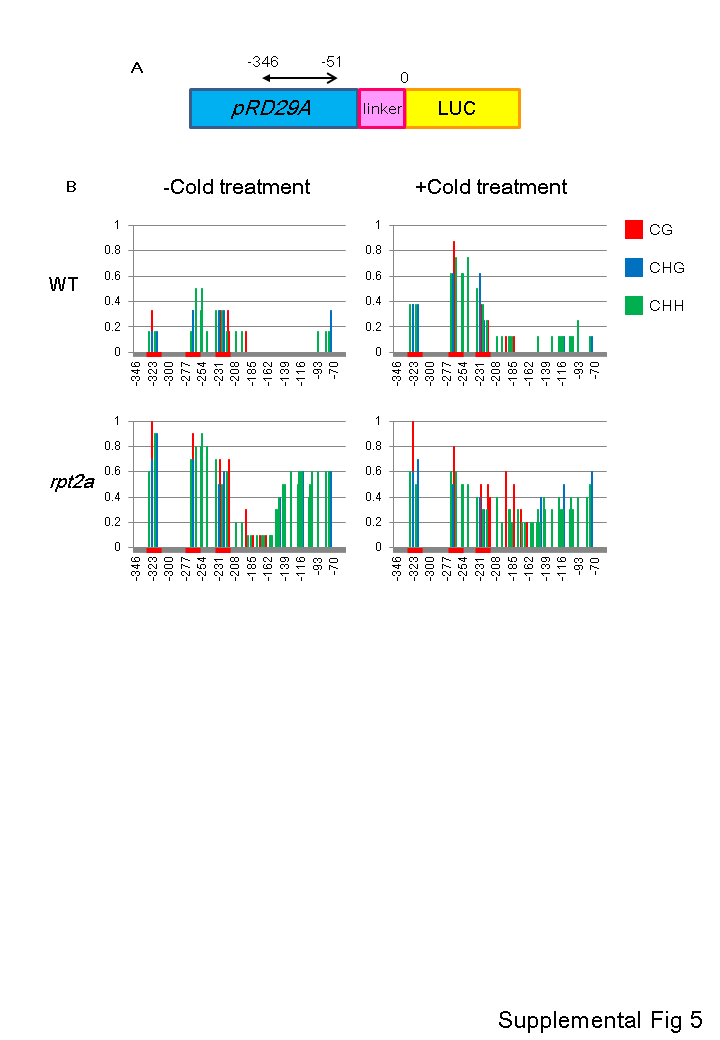

Supplement: Figure S5 — (A) Scheme of analyzed region in exogenous RD29A promoter. (B) Bisulfite sequencing of DNA methylation in the exogenous RD29A promoter site (from −346 bp to −51 bp upstream of the promoter). Upper graph shows methylation status in WT and the lower graph shows DNA methylation status in the rpt2a-2 mutant. The height of the vertical lines shows the frequency of methylcytosine. Red, blue and green lines indicate frequencies of methylcytosine at CG, CHG and CHH sites, respectively. Red bars on the x-axis are DRE and DRE/CRT core sequences. Twenty clones are sequenced for each sample. (TIF) [file pone.0037086.s005.tif]

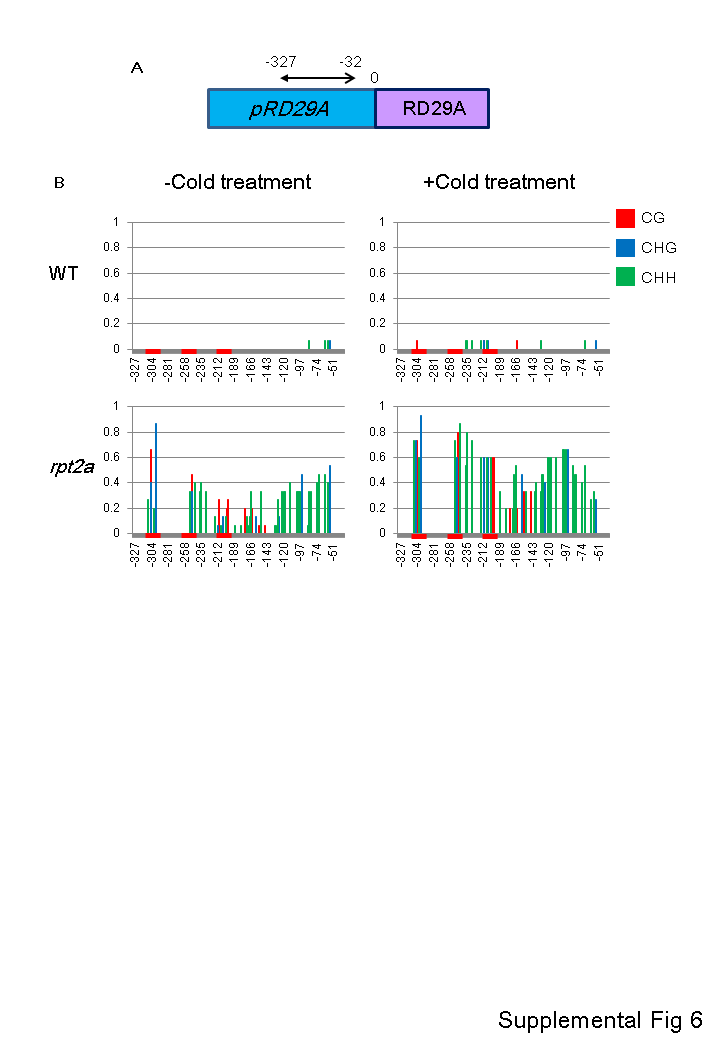

Supplement: Figure S6 — (A) Scheme of analyzed region in endogenous RD29A promoter. (B) Bisulfite sequencing of DNA methylation in the endogenous RD29A promoter site (from −327 bp to −32 bp upstream of promoter). The upper graph shows methylation status in WT and the lower graph shows DNA methylation status in the rpt2a-2 mutant. The height of the vertical lines shows the frequency of methylcytosines. Red, blue and green lines indicate frequencies of methylcytosine at CG, CHG and CHH sites, respectively. Red bars on the x-axis are DRE and DRE/CRT core sequences. Twenty clones are sequenced for each sample. (TIF) [file pone.0037086.s006.tif]

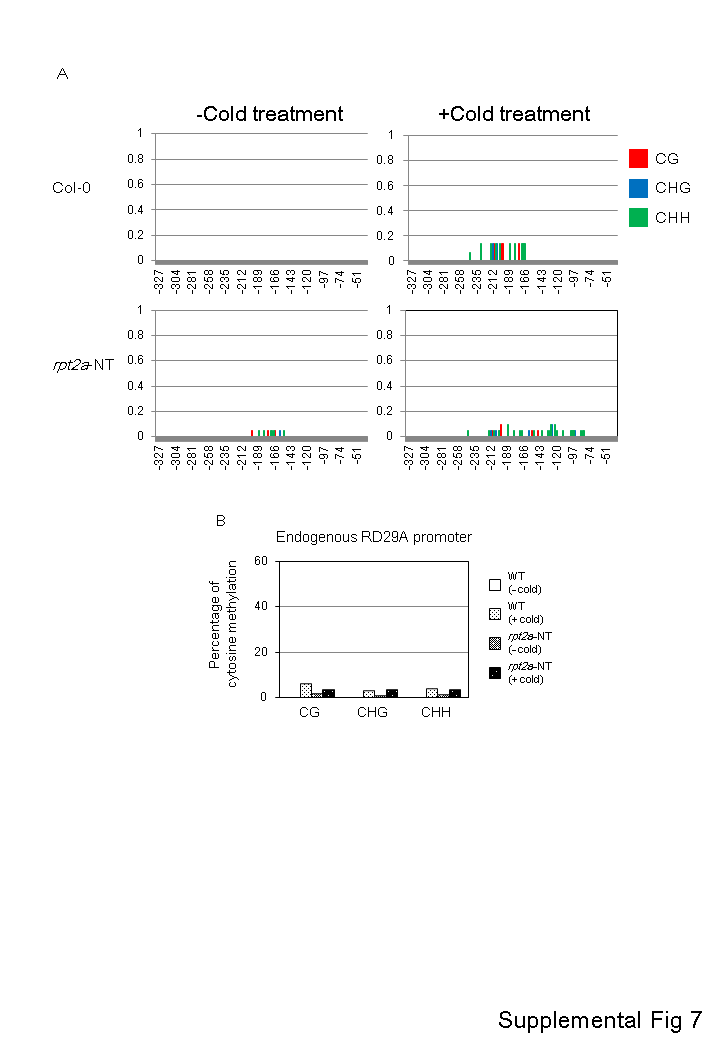

Supplement: Figure S7 — (A) Bisulfite sequencing of DNA methylation in the endogenous RD29A promoter site (from −327 bp to −32 bp upstream of the promoter) in Col-0 and rpt2a-2 (no transgene: rpt2a-NT). The upper graph shows methylation status in cold-untreated and treated Col-0, and the lower graph shows cold-untreated and treated rpt2a-2 (no transgene). (B) Mean levels of DNA methylation in different cytosine context at the exogenous and endogenous RD29A promoter in Col-0 and rpt2a-2 (no transgene). Red, blue and green lines indicate frequencies of methylcytosine at CG, CHG and CHH sites, respectively. Twenty clones are sequenced for each sample. (TIF) [file pone.0037086.s007.tif]

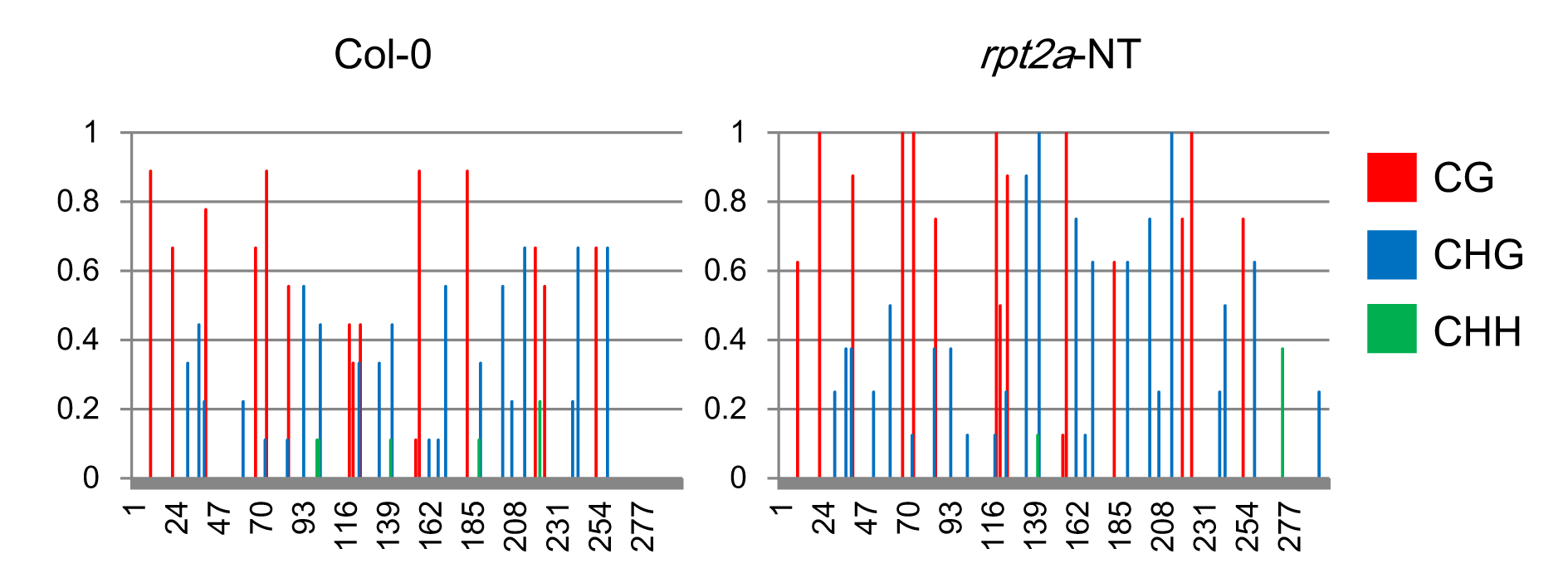

Supplement: Figure S8 — Bisulfite sequencing of DNA methylation in the AtGP1 site in Col-0 and rpt2a-2 (no transgene: rpt2a-NT). (TIF) [file pone.0037086.s008.tif]

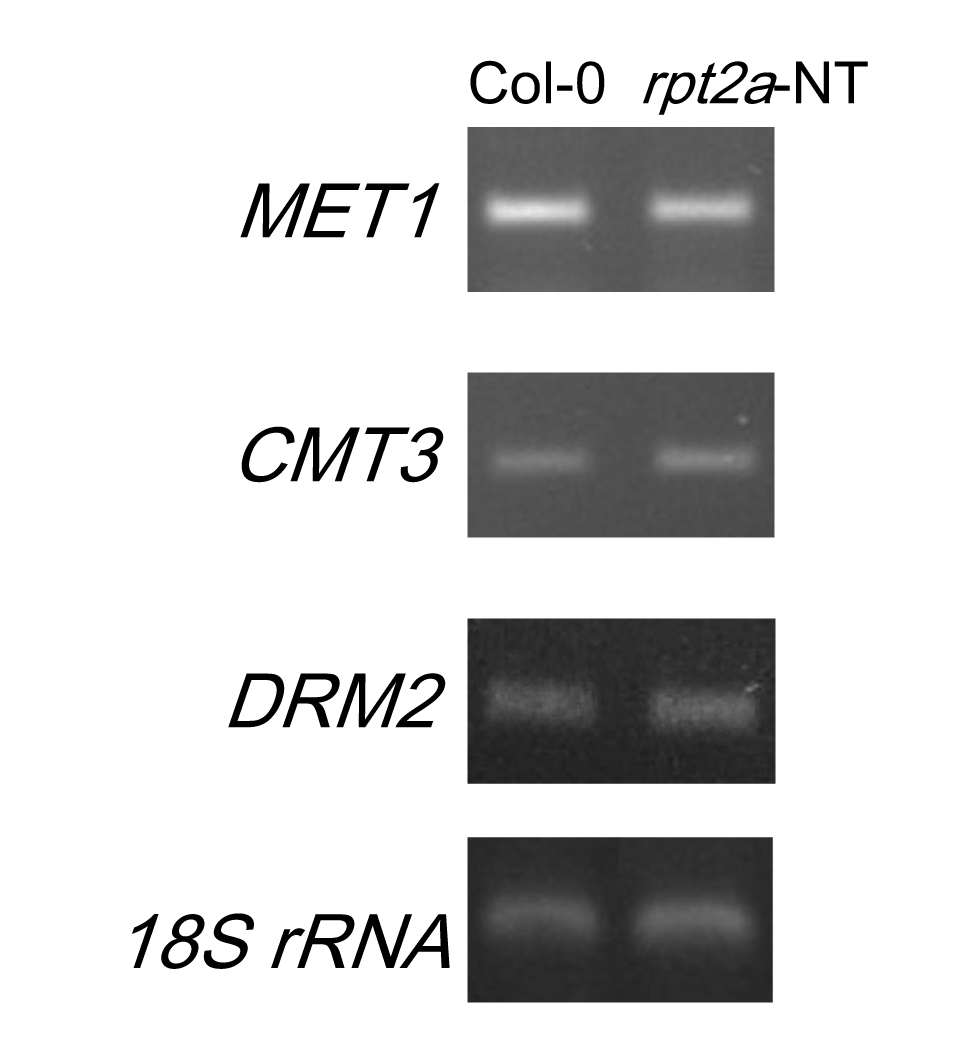

Supplement: Figure S9 — RT-PCR analysis of DNA methyltransferase genes: MET1, CMT3, DRM2 and 18S rRNA (control). (TIF) [file pone.0037086.s009.tif]
