# Supplementary material for: Arabidopsis RPT2a, 19S Proteasome Subunit, Regulates Gene Silencing via DNA Methylation
Source: PLoS One. 2012 May 16;7(5):e37086. doi: 10.1371/journal.pone.0037086 (PMC3353898; doi:10.1371/journal.pone.0037086)
Supplement: Table S1 — Primers used in this study. (PDF) [file pone.0037086.s010.pdf]

Table S1 primer list

purpose	primer name	sequences
For Real time PCR	*LUC of 35S F*	CCACGCTGGGCTACTTGATC
	LUC of 35S R	TCCTCCTCGAAGCGGTACAT
	LUC of RD29A F	GCGACCAACGCCTTGATT
	LUC of RD29A R	TCCCAGTAAGCTATGTCTCCAGAA
	RD29A F	TGATCGATGCACCAGGCGTAAC
	RD29A R	CCCTGGTGGAATAATTTCCTCCG
	18S rRNA F	CGGCTACCACATCCAAGGAA
	18S rRNA R	GCTGGAATTACCGCGGCT
For Bisulfite sequencing	*pRD29A nested*	GTAAATGTAAAATGATTATATGATGGG
	pRD29A converted	GGATCGATTATTAATAATAGTAAGTTAT
	pRD29A exo R1	ATTCTATAATTTATATTCAACCCATATC
	pRD29A exo R2	AAATACTCAACATAAATAATATCCACC
	pRD29A endo R1	CTAAAATTAAAATCTACCTAAATACTAC
	pRD29A endo R2	ATAATAATTCCTCTATTTAATCCATTTTCC
	AtGP1 F1	TATAGTGTTATAGTTGAGTAGAGTG
	AtGP1 F2	TTGTAGTTTGAGTTTGTGGAGTTTG
	AtGP1 R	CAAAAAAATACTCAATACCAATCCTCTA
	AtMEA-ISR F	AAAGTGGTTGTAGTTTATGAAAGGTTTTAT
	AtMEA-ISR R	CTTAAAAAATTTTCAACTCATTTTTTTTAAAAAA
For Mcr-PCR	*AtSIN F*	ACTTAATTAGCACTCAAATTAAACAAAATA
	AtSIN R	TTTAAACATAAGAAGAAGTTCCTTTTTCAT
	AtGP1 F	ACAGTGCCACAGTTGAGCAG
	AtGP1 R	CAGAAAAATACTCGGTGCCAAT
	AtMu1 F	GTGGATATACCAAAAACACAA
	AtMu1 R	CTTAGCCTTCTTTTCAATCTCA
	AtMEA-ISR F	TCCAACATGTTTTTCACATCTTC
	AtMEA-ISR R	GGTTATGAATGAAGTATAGTTAG
	AtCOPIA4 F	CTCACTCAAGCTTCGGTTCC
	AtCOPIA4 R	TGTTGGTGAAGGACCGTACA
	ACTIN2 F	AAACCTCAAAGACCAGCTCTT
	ACTIN2 R	AACGATTCCTGGACCTGCC
For expression analysis	*COR15A F*	AGCGGAGCCAAGCAGAGCAG
	COR15A R	TGCGGCTTCTTTTCCTTTCTCC
	DREB1b F	CGGCGGGCCGTAAGAAGTTT
	DREB1b R	GACGGCGGCGGTAAAAGCAT
	MET1 5'	ATCTGCAAGGCTATGAATGAGC
	MET1 3'	CTTCTTGAGATGTAGGGCTTCT
	CMT3 5'	AGGGACTTTCCTGGTGTTATTG
	CMT3 3'	TGCAAGCTCGGAAGGAAGAGTTGGC
	DRM2 5'	GAAGTGGCACTTCATCGTCTC
	DRM2 3'	AGATCCTCTCATCCTCGCACGT
	ABI3 5'	GTGGCAAGTGTTGATCATGG
	ABI3 3'	CGTGAAAACCGTCCAAATCT
	At3g29650 5' TE	AAAGGAATGATTCGCCTGGC
	At3g29650 3' TE	TCAAACTCTGGCTTCACAGGTC
For Southern blot	*LUC2 5'*	ATGGAAGATGCCAAAAACAT
	LUC2 3'	GAAGTCGTACTCGTTGAAGC
	LUC 5'	ATGGAAGACGCCAAAAACATAAAG
	LUC 3'	CCCTTTTTGGAAACGAACACCAC
TAIL PCR	LB-3 for pGWB	CGTCCGCAATGGTTATTAAGT
	LB-2 for pGWB	TTGCCGCCGATCTTCATAGGG
	LB-1 for pGWB	CCACCTCACCTTGGGTTCCTT
	LB-3 for pGWB	ATGGTTCACGTAGTGGGCCATCG
	LB-2 for pGWB	AGTGGACTCTTGTTCCAAACTGG
	LB-1 for pGWB	CAAACCAGCGTGGACCGCTTGCTGCAAC
For insertion check	*LP for 35S::LUC2*	ATGAGTGAAGAAGACGGCGGCT
	RP for 35S::LUC2	TCTAACCTCTGCTTCTTCTTCG
	LB for 35S::LUC2	CGTCCGCAATGGTTATTAAGT
	LP for RD29A::LUC	ATGTTCGAATAACCATAGATGAT
	RP for RD29A::LUC	TGAAAATATCCACAGTTCCCAT
	LB for RD29A::LUC	GCGTGGACCGCTTGCTGCAACT
