# Supplementary material for: Arabidopsis RPT2a, 19S Proteasome Subunit, Regulates Gene Silencing via DNA Methylation
Source: PLoS One. 2012 May 16;7(5):e37086. doi: 10.1371/journal.pone.0037086 (PMC3353898; doi:10.1371/journal.pone.0037086)
Supplement: Methods S1 — Supplementary methods. (PDF) [file pone.0037086.s011.pdf]

Methods S1

Southern blot analysis

DNA extracted from 2-week-old Col-0, 35S::LUC2 in WT and in rpt2a plants (10 mg), was incubated overnight with EcoRI. The DNA was transferred to a nitrocellulose membrane and probed with AlkPhos Direct labeled (GE) LUC2 gene nucleic acid sequence. The chemiluminescent signal was detected with CDP-STAR detection reagent (GE). DNA extracted from 2-week-old Col-0, RD29A::LUC in WT and in *rpt2a* plants (3 mg), was incubated overnight with SacI. The DNA was transferred to a nitrocellulose membrane and probed with AlkPhos Direct labeled (GE) LUC gene nucleic acid sequence. The chemiluminescent signal was detected with CDP-STAR detection reagent (GE).

TAIL-PCR

DNA extracted from 2-week-old 35S::LUC2 in WT plants and RD29A::LUC in WT plants and used as template. Three rounds of PCR were carried out using the PCR primers listed in Supplemental Table 1 and random primers. The PCR products were cloned into a pCR2.1 vector (Invitrogen) and clones were subjected to sequence analysis.
